# Mechanobiological Response of Peri-Implant Bone to Variations in Inter-Implant Distance: A Finite Element Analysis of Conometric Implants at Crestal and Subcrestal Positions

**DOI:** 10.3390/jfb17050208

**Published:** 2026-04-28

**Authors:** Mario Ceddia, Tea Romasco, Natalia Di Pietro, Luciano Lamberti, Bartolomeo Trentadue

**Affiliations:** 1 Department of Mechanics, Mathematics and Management, Polytechnic University of Bari, 70125 Bari, Italy; marioceddia1998@gmail.com (M.C.); luciano.lamberti@poliba.it (L.L.); bartolomeo.trentadue@poliba.it (B.T.); 2 Department of Medical, Oral and Biotechnological Sciences, “G. D’Annunzio” University of Chieti-Pescara, 66100 Chieti, Italy; tea.romasco@unich.it

**Keywords:** internal connection, bone loss, inter-implant distance, FEA

## Abstract

Inter-implant distance (IID) is crucial for peri-implant bone preservation and long-term implant success. Traditionally, a minimum IID of 3 mm is recommended to limit marginal bone loss, although the biomechanical effect of smaller distances remains debated and may depend on multiple biological, prosthetic, and surgical factors. This study uses finite element analysis (FEA) to evaluate the effect of IID on stress distribution in peri-implant bones of D3 and D4 quality, considering crestal versus subcrestal implant placement, and interpreting results within Frost’s mechanostat theory. Implants with an internal conometric connection were modeled within simulated D3 and D4 mandibular bone blocks. IID values of 3 mm, 1.5 mm, and 1 mm were analyzed under masticatory load. Von Mises stresses in cortical and trabecular bone were compared against biomechanical thresholds (2 MPa disuse and 20 MPa remodeling limit). Results: Cortical stress increased with decreasing IID, more pronounced in crestal placement. In D3 bone, maximum cortical stress rose from 7.2 MPa (3 mm IID) to 16.5 MPa (1 mm IID) under crestal placement, while remaining within the mechanostat-based thresholds adopted in the present stress-interpretation framework. In D4 bone, cortical stress approached 20 MPa at 1 mm IID under crestal placement, indicating a less favorable mechanical condition within the interpretive framework adopted. Subcrestal placement reduced cortical stresses in both bone qualities. Trabecular stress remained stable in D3 (~1.7–8 MPa) and increased moderately in D4 (~up to 13 MPa). Conclusions: Within the limitations of this preclinical finite element study, decreasing inter-implant distance was associated with increased cortical stress, while subcrestal placement was associated with lower cortical stress than crestal placement. These findings should be interpreted only as comparative computational results, and no direct clinical conclusion can be drawn regarding the acceptability of a 1 mm inter-implant distance.

## 1. Introduction

Dental implants have a long-term success rate, which has led to their widespread use in a multitude of clinical applications. Since osseointegrated implants were introduced for edentulous patients in the late 1960s and Brånemark defined the clinical protocols, awareness of, and demand for fixed prostheses has steadily increased [1,2,3,4,5,6].

Recent developments have demonstrated that implant-supported fixed prostheses are a widely accepted alternative to removable dentures in cases of partial or total tooth loss, including challenging situations such as severe mandibular or maxillary atrophy [7,8].

Implant failure may be influenced by both biological and mechanical factors. Poor bone quality, previous periodontitis, and inadequate oral hygiene may increase the risk of peri-implant disease and marginal bone loss [9,10,11,12], while occlusal overload and unfavorable prosthetic conditions may promote stress concentration and micromovements at the bone–implant interface, thereby impairing osseointegration and long-term stability [13,14,15]. Conversely, bone maintenance may be favored when the mechanical stimulus remains within a physiological range [16].

When planning adjacent implants, inter-implant distance (IID) is a key factor because it influences peri-implant tissue morphology and bone support. In particular, reduced spacing has been associated with greater marginal bone loss, and sites with an IID of less than 3 mm generally show more crestal bone resorption than those with an IID of 3 mm or more [17,18,19,20,21].

Clinical and histological studies have suggested operative thresholds to minimize crestal bone resorption. For example, Saadoun et al. [22] recommend supporting a minimum distance of 2 mm between an implant and a natural tooth and 3 mm between two adjacent implants. This is intended to reduce crestal bone loss (>1.5 mm), promote soft tissue fill, and support the interdental papilla. Over two decades ago, Tarnow et al. [23] highlighted the importance of preserving a distance of at least 3 mm between adjacent implants, reporting loss of inter-implant crestal bone when this distance was not kept. Later studies have also investigated the ideal inter-implant distance (IID). For instance, Traini et al. [24] found that an IID of 3 mm was associated with greater vascularization than an IID of 2 mm, showing a more suitable environment for angiogenesis and osteogenesis. It should be noted that many of the observations reported in the literature are based on implants with an external hexagonal connection. More recent studies have investigated whether these recommendations also apply to implants with platform switching and internal conical/conometric connections, reporting generally favorable long-term outcomes [25,26,27,28]. Histological and clinical evidence has suggested that these systems may support crestal bone preservation, high bone-to-implant contact, and reduced bacterial microleakage, thereby contributing to a favorable peri-implant tissue response [29,30,31,32,33,34,35].

Although traditional guidelines recommend an IID of at least 3 mm, Schwarz et al. [36] reported stable crestal bone levels with an IID of 1 mm and a bone level found coronal to the implant shoulder in a clinical case with a 10-year follow-up. The authors attributed this outcome to various factors, such as slightly subcrestal placement, platform switching and a rough implant surface that promoted anchorage, as well as the stability provided by the internal conometric connection. Together, these factors may have contributed to bone preservation, even when the IID was lower than the 3 mm recommended by historical evidence.

Despite the large number of available studies, an ‘optimal’ IID that ensures adequate biomechanical support in relation to bone density and vertical implant position (crestal versus subcrestal) has not yet been defined [36]. Furthermore, the precise placement of implants may be subject to positional inaccuracies, including deviations in inter-implant distance.

From a clinical perspective, the planned inter-implant distance does not always coincide with the distance achieved during surgery. Positional deviations may occur even when the operator tries to maintain predefined mesiodistal spacing, and this aspect may become particularly relevant when reduced inter-implant distances are planned. Zadrożny et al. [37] highlighted that repeatability and precision are important determinants of the final implant position, showing that freehand implant placement, even when assisted by universal plastic sleeves, may still be associated with measurable deviations from the intended site. Therefore, surgical accuracy should be regarded as an additional factor that may influence the actual inter-implant distance and, so, the biomechanical environment of the inter-implant bone crest.

In this context, finite element analysis (FEA) has become a widely used and reliable computational tool for investigating the biomechanical behavior of dental implant systems under different clinical conditions, including variations in inter-implant distance. Its major strength lies in the ability to reproduce complex implant geometries, heterogeneous material properties, and clinically relevant boundary conditions that are often difficult to assess experimentally. Accordingly, FEA has been extensively adopted in implant dentistry to predict stress and strain distribution within both implant components and peri-implant bone, thus offering useful insight into the mechanical factors that may influence treatment outcomes.

From a methodological standpoint, FEA is based on the construction of a mathematical model of the structure of interest, which is discretized into a finite number of small, interconnected elements. The mechanical response of the system is then solved numerically by calculating stress, strain, and displacement fields at the nodal points, thereby allowing a detailed evaluation of load transfer mechanisms within the implant–bone complex.

FEA studies by Ceddia et al. [38,39] showed that stress concentrations in the cortical bone are reduced by subcrestal placement (approximately −2 mm) for a single-implant restoration with a conometric connection compared with equicrestal placement. Furthermore, bone quality significantly affects stress transfer from the implant to the bone: higher cortical stress peaks and a reduction in the implant stability quotient (ISQ) were seen in low-density bone.

Beyond purely static analyses, FEA has also been coupled with bone-remodeling algorithms based on Frost’s mechanostat to simulate the temporal evolution of bone density around implants. In these approaches, a local mechanical stimulus (e.g., stress, strain or strain energy density) computed from FEA is used as input to a remodeling law that updates the clear density or elastic modulus of each element iteratively until a quasi-steady state is reached [40]. Pioneering works by Huiskes et al. [41] applied adaptive bone-remodeling theory in combination with the finite element method to prosthetic design analysis, proving the feasibility of predicting stress-driven bone adaptation. More recent studies have implemented mechanostat-based remodeling models around osseointegrated dental implants in the mandible, showing that these simulations can reproduce clinically observed patterns of peri-implant bone loss and apposition and can be used to assess the long-term biomechanical performance of different implant configurations and loading conditions [42,43].

The present study aimed to use finite element analysis (FEA) to evaluate the effect of inter-implant distance (IID) on stress transmission to the surrounding peri-implant bone in relation to bone density, vertical implant position with respect to the bone crest (crestal versus subcrestal), and conometric implant connection, and to interpret the computed stress values in light of Frost’s mechanostat theory.

## 2. Materials and Methods

### 2.1. Modeling

The CAD models of the implants were developed using Autodesk Inventor 2023 and reproduce an internal conometric implant–abutment connection. Each implant is 13 mm in length and 3.5 mm in diameter, while the abutment length is 8.2 mm. The implants feature a flat apex, designed to reduce bone trauma during insertion, improve positional accuracy, and enhance primary stability.

In the present study, a simplified standardized bone model was adopted instead of a patient-specific CT/CBCT-derived geometry to isolate the effects of inter-implant distance, bone quality, and vertical implant position under controlled conditions. Although patient-specific image-based models may reproduce anatomical details better, they also introduce more uncertainty related to image segmentation and to the assignment of mechanical properties from radiological data, particularly when CBCT gray values are affected by artifacts and scanner-dependent variability. Therefore, a simplified mathematical model was considered more right for the comparative purpose of this preclinical investigation.

A representative mandibular bone block was constructed with a cortical thickness of 1.0 mm for D3 bone and 0.5 mm for D4 bone, according to Misch’s classification [44]. This classification defines bone quality based on apparent density: D1, very dense cortical bone (≳1250 HU); D2, dense/porous cortical bone with high-density trabecular bone (850–1250 HU); D3, thin/porous cortical bone with low-density trabecular bone (350–850 HU); D4, very thin cortical bone with low-density trabecular bone (150–350 HU); and D5, immature or regenerated bone (<150 HU). Clinically, D3–D4 bone is associated with reduced primary stability and often requires under-preparation of the implant site and longer healing periods, whereas D1–D2 bone generally provides higher primary stability but requires careful drilling to minimize thermal injury [45].

**Figure 1:**
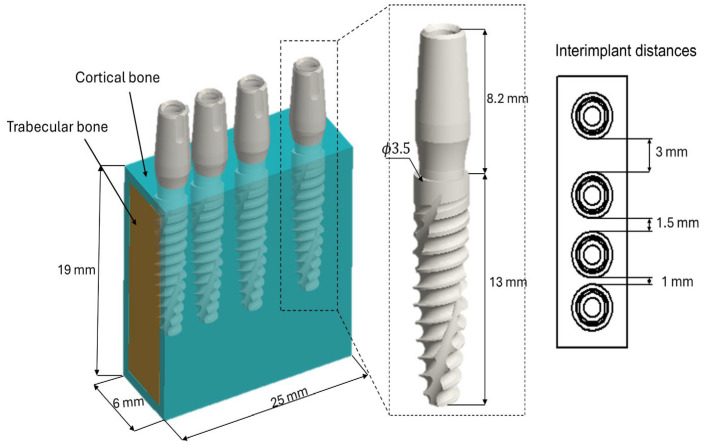
3D model of the implant–bone system showing the geometric dimensions of the bone block, the implant, and the distance between implants.

The implants were positioned either at the crestal level or 2 mm subcrestally (−2 mm relative to the bone crest) and arranged in pairs with neck-to-neck inter-implant distances of 3.0 mm, 1.5 mm, and 1.0 mm (Figure 1).

**Figure 2:**
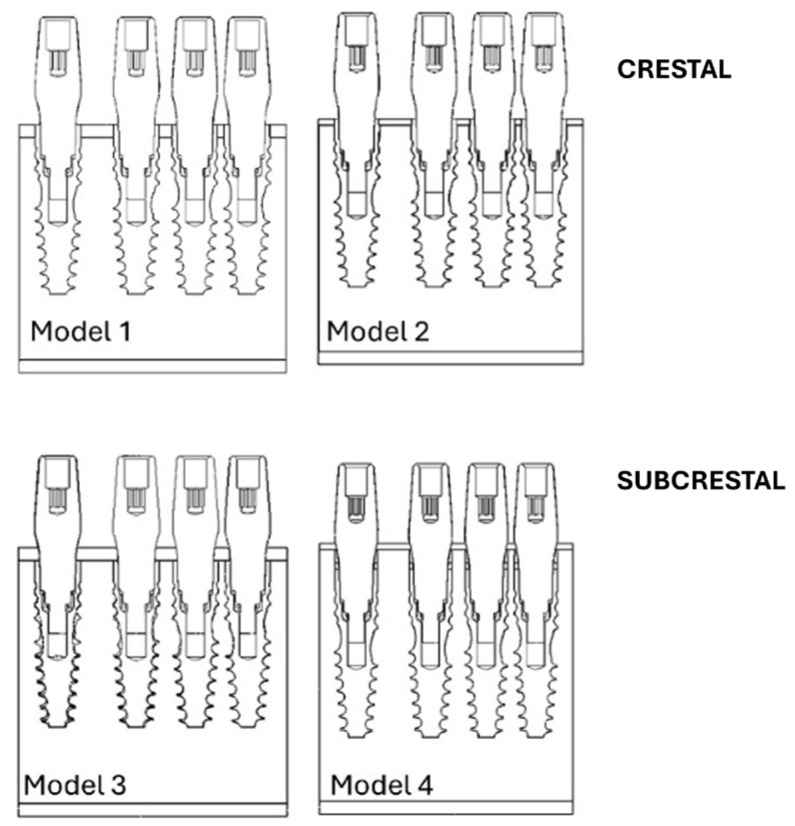
Models analyzed in this study, categorized by bone class (D3–D4) and implant placement (crestal versus subcrestal).

Figure 2 illustrates the four finite element models analyzed in this study:

- Model 1: implants placed at the crestal level in D3 bone.
- Model 2: implants placed at the crestal level in D4 bone.
- Model 3: implants placed at the subcrestal level in D3 bone.
- Model 4: implants placed at the subcrestal level in D4 bone.

The mechanical properties of bone are a key prerequisite for the biomechanical analysis of dental implants. Alveolar bone consists of a low-density trabecular bone, arranged in a three-dimensional network of trabeculae, and a denser cortical layer (lamina dura), which absorbs and transfers most of the masticatory loads. The trabecular bone contributes to distributing the stress fields within the bone volume, attenuating peak stresses, and reducing the risk of local concentrations that could lead to tissue damage.

From a mechanical standpoint, bone is an anisotropic, heterogeneous, and porous material due to its internal structure and the presence of marrow cavities. However, numerous finite element studies have shown that, under physiological loading and small strain levels, its behavior can be accurately approximated as that of a linear elastic, isotropic material (and often treated as homogeneous) [46]. This assumption allows the use of a single elastic modulus and Poisson’s ratio in numerical modeling.

Table 1 reports the mechanical properties of trabecular and cortical bone for D3 and D4 bone types, respectively. The implant and abutment were modeled as a titanium alloy (Ti-6Al-4V) with a Young’s modulus of 110 GPa and a Poisson’s ratio of 0.3 [47].

### 2.2. FEA Modeling

Using the finite element software ANSYS Workbench R2023, the three-dimensional model of the bone block and implants was discretized with tetrahedral elements, selected for their ability to accurately capture stress and strain distributions in complex geometries.

A mesh sensitivity analysis was then performed to determine the best element size. Reducing the element size from 1.0 mm to 0.85 mm produced substantial changes in the stress values, particularly in the region of the implant neck, while further refinement to 0.5 mm resulted in differences of less than 1%. This was taken as evidence of mesh convergence.

**Figure 3:**
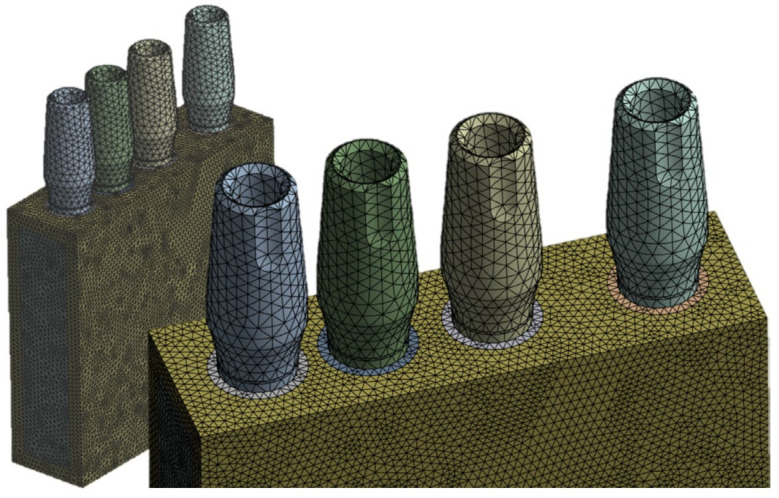
Discretized model with tetrahedral elements, with a mesh size of 0.5 mm.

Consequently, a 0.5 mm element size was adopted as the best configuration, providing a balance between computational efficiency and numerical accuracy. The final mesh consisted of 17,539 elements and 36,851 nodes (Figure 3).

Subsequently, two contact interfaces were defined. A bonded contact was assigned at the implant–bone interface to simulate complete osseointegration, while a frictional contact with a static friction coefficient of 0.25 was defined between the abutment and the implant.

### 2.3. Boundary Conditions

**Figure 4:**
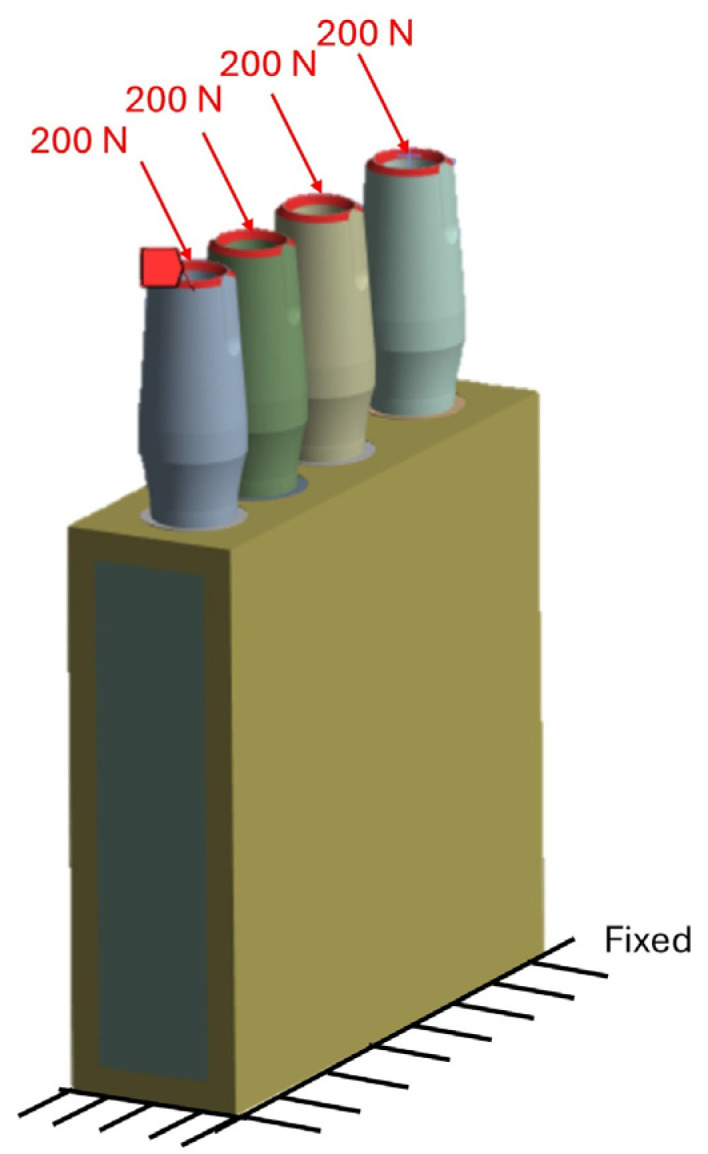
Loading and boundary conditions.

In this study, loads were applied directly to the implants, rather than through the prosthetic superstructure, to simplify the model and isolate the specific effect of inter-implant distance on peri-implant bone stress. Although this approach does not fully reproduce clinical loading conditions, it was considered acceptable for comparative purposes, since the regions of interest were located away from the load application area and, according to Saint-Venant’s principle, local stress concentrations near the point of load application were not expected to substantially influence the overall bone stress patterns. An oblique load of 200 N applied at 30° was selected to represent a standardized moderate posterior functional loading condition commonly used in dental implant finite element studies, rather than a maximum bite force, which is highly variable among individuals. This loading scenario is still clinically relevant, as oblique forces are generally more critical than axial loads in transmitting stress to the peri-implant bone [48]. The supporting bone block was fully constrained in all directions to ensure model stability (Figure 4), and a static structural analysis was performed to evaluate load transfer from the implants to the surrounding bone and to identify regions potentially exposed to elevated stress and excessive bone remodeling.

The average computational time for each model was approximately 15 min, using a system equipped with two 13th-generation Intel^®^ Core™ i7 processors, Intel Corp., Santa Clara, CA, USA (14 cores each; 4 CPU units in total) and 16 GB of RAM.

In the present study, von Mises stress was used as a scalar indicator to compare the mechanical state of the peri-implant bone among the different configurations analyzed. Although bone is not a ductile isotropic material, von Mises stress is widely adopted in dental finite element studies as a comparative parameter for showing regions of stress concentration and for evaluating the relative influence of design and loading variables under consistent modeling assumptions.

### 2.4. Stress Interpretation According to Frost’s Mechanostat

**Figure 5:**
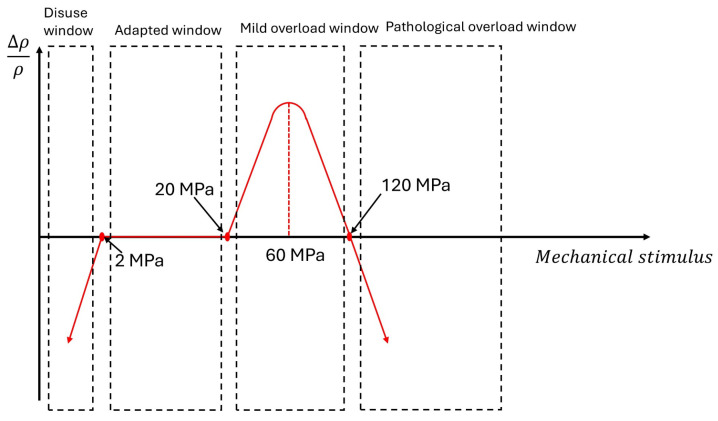
Frost’s mechanostat windows are based on the bone density change ($\frac{\Delta \rho }{\rho }$) as a function of the mechanical stimulus.

In numerical models, the mechanobiological behavior of bone is often described in terms of “stress windows” (Figure 5).

In the present study, no bone remodeling simulation was performed. Instead, the von Mises stress values computed in the peri-implant bone were interpreted according to Frost’s mechanostat theory, using stress thresholds as a biomechanical reference framework. A lower threshold of 2 MPa and an upper threshold of 20 MPa were considered to identify stress levels associated with underloading and overload, respectively. Therefore, the present analysis provides a stress-based interpretation of the possible biomechanical environment around the implants, rather than a direct simulation of time-dependent bone remodeling.

## 3. Results

**Figure 6:**
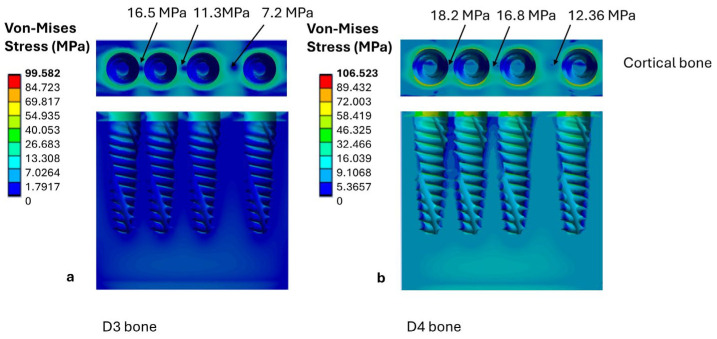
Von Mises stress distributions in the cortical and trabecular bone for crestal implant placement as a function of bone quality: (**a**) D3 bone; (**b**) D4 bone.

It is shown in Figure 6 that the maximum stresses occur in the cortical bone. For D3 bone, cortical stress varies according to the distance between implants: the peak stress is 7.2 MPa at an IID of 3.0 mm, increasing to 11.3 MPa at 1.5 mm, and reaching 16.5 MPa at 1.0 mm (Figure 6a). Therefore, reducing the distance between implants results in an increase in cortical stress. However, these values remained within the mechanostat-based reference window adopted in the present study (Figure 5). Within this computational framework, this finding indicates a lower relative likelihood of overload compared with configurations showing higher stress values, but should not be interpreted as evidence of biological safety or clinical acceptability.

In the trabecular portion of the D3 bone, stress remains almost insensitive to changes in IID, remaining constant at around 1.7–2.0 MPa across configurations.

By contrast, D4 bone shows higher stress levels, reflecting its reduced mechanical strength. In the cortical layer, the maximum stress is 18.2 MPa when the implants are 1.0 mm apart (Figure 6b). In the trabecular bone, stress also increases with decreasing IID, attaining values between 5.5 and 10 MPa. Nevertheless, even in low-density D4 bone, cortical stress values remained within the mechanostat-based thresholds considered in the present study; this should be regarded only as a comparative computational finding within the adopted interpretive framework.

**Figure 7:**
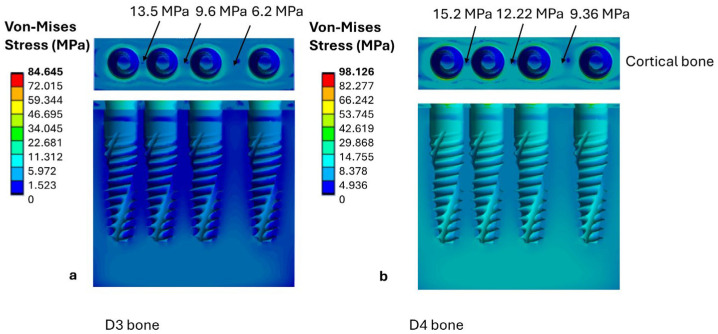
Von Mises stress distribution in the cortical and trabecular bone for subcrestal implant placement as a function of bone quality: (**a**) D3 bone; (**b**) D4 bone.

Subcrestal implant placement reduced the stresses transmitted to the cortical bone in both D3 and D4 bone classes. As shown in Figure 7a, in D3 bone, the maximum cortical stress decreased from 13.5 MPa to an inter-implant distance of 1 mm to 6.2 MPa at 3 mm. A similar trend was seen in D4 bone, where the maximum cortical stress decreased from 15.2 MPa at 1 mm to 9.36 MPa at 3 mm, as shown in Figure 7b.

Although the stress values in D4 bone remained higher than those recorded in D3 bone, they still fell within the stress interval defined by the mechanostat-based reference thresholds adopted in this study; this observation should not be interpreted as proof of physiological safety.

**Figure 8:**
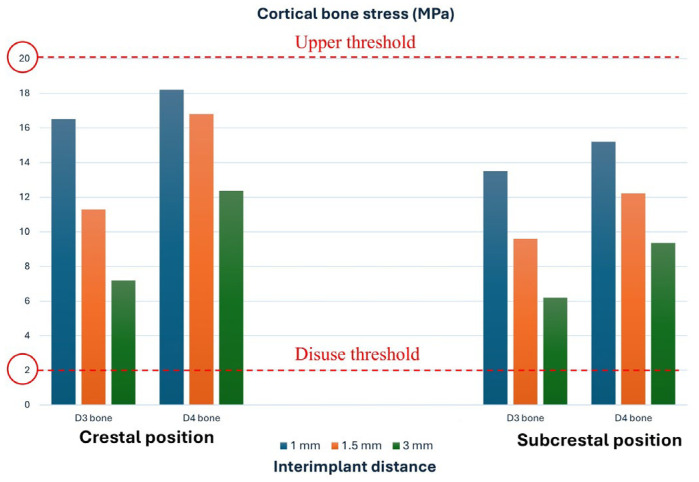
Cortical bone stress as a function of inter-implant distance, bone quality (D3–D4), and crestal/subcrestal position, with mechanostat disuse and upper remodeling thresholds.

Figure 8 summarizes the cortical bone stress values for both D3 and D4 bone. These values remained within the two mechanostat-based thresholds considered in this study, namely 2 MPa and 20 MPa. Within this reference framework, the computed stresses were interpreted as belonging to a range compatible with physiological loading rather than pathological overload. In D4 bone, especially under crestal placement and 1 mm IID, the stress values approached the upper threshold, showing a less favorable mechanical condition. However, no configuration exceeded the overload threshold adopted in the present stress-based interpretation.

For D3 bone, reducing the inter-implant distance from 3.0 to 1.0 mm resulted in a progressive increase in cortical stress, which was more pronounced for crestal placement than for subcrestal placement. D4 bone showed systematically higher stress values, with the crestal 1.0 mm IID configuration approaching the upper threshold considered in the present framework. Nevertheless, none of the analyzed configurations exceeded the upper threshold or fell below the lower threshold adopted for stress interpretation within the present computational framework.

In summary, within the IID range investigated (1–3 mm), no configuration showed cortical stress values below the lower mechanostat-based threshold adopted in the present study. This should be interpreted only as a comparative computational observation and not as a direct prediction of underloading-related bone resorption.

## 4. Discussion

It is considered essential to follow implant placement recommendations, as it is believed that this will have a positive effect on the long-term maintenance of peri-implant tissues. Two decades ago, Tarnow et al. [23] established guidelines for ensuring correct mesio-distal implant placement. Numerous clinical studies have confirmed that an inter-implant distance (IID) of less than 3 mm is associated with significant bone loss [49,50,51,52].

Recent retrospective studies and systematic reviews have shown that inter-implant distances of less than 3 mm are associated with increased marginal bone loss and a higher likelihood of biological complications in the medium to long term, especially in the presence of conventional connections and in posterior sites subjected to high functional loads. De Angelis et al. [53], in a literature review, reported that reduced horizontal distances between implants are consistently associated with greater crestal bone resorption compared with IIDs ≥ 3 mm. Similarly, Toia et al. [54] observed that inter-implant spaces of less than 3 mm correlate with increased bone loss, emphasizing that the critical threshold may vary according to the type of implant–abutment connection and the surgical protocol adopted.

Other clinical studies have reported a higher incidence of resorption of the inter-implant crest and soft tissue recession when implants are placed too close together, with consequent deterioration of radiographic parameters and, in some cases, the need for corrective interventions or fixture replacement [54,55,56,57]. Cardaropoli et al. [58] documented significant peri-implant alterations related to the distance between implant units, confirming the critical role of three-dimensional geometry in long-term bone stability. Other authors have concluded that inter-implant distances of less than 3 mm between adjacent implants lead to increased crestal bone loss and a reduction in the height of the inter-implant crest, with possible repercussions for the interdental papilla, esthetic outcomes, and the overall implant prognosis [59].

However, preclinical and clinical studies comparing bone responses at 2 mm versus 3 mm between implants have repeatedly shown that an IID of 2 mm does not compromise preservation of the crestal bone between implants, particularly when features such as platform switching and internal connections are present [60,61]. Moreover, two experimental studies comparing an inter-implant distance (IID) of 1 mm with larger distances reported a more favorable bone response at the shorter spacing [62,63,64,65]. In addition to the reduced angiogenesis associated with implants placed too close together, as seen by Traini et al. [64], mechanical loading also plays a key role in peri-implant bone resorption.

However, the biomechanical response seen in the present model should not be interpreted as the sole determinant of clinical success. Although an IID of 1 mm remained within the mechanostat thresholds considered in this simulation, reduced inter-implant spacing may still entail relevant biological and clinical risks that were not directly reproduced in the finite element analysis. First, a narrower inter-implant space may compromise vascularization of the interproximal bone crest, thereby potentially affecting angiogenesis and long-term crestal bone maintenance. Second, the reduced safety margin observed in the D4 crestal configuration, where cortical stress approached the upper remodeling threshold, suggests that in vivo factors such as occlusal imbalance or parafunctional habits could more easily shift the local mechanical environment toward overload-related bone resorption. Third, from a surgical perspective, planning very small inter-implant distances requires high positional accuracy, since even minor deviations may reduce the effective spacing or, in extreme cases, result in unwanted proximity between implant bodies. Therefore, the mechanical response observed for the 1 mm IID configuration under the assumptions of the present model should be interpreted within a broader multifactorial framework that also includes biological, prosthetic, and technical considerations.

In this regard, Zadrożny et al. [37] reported that freehand implant placement, even when assisted by universal plastic sleeves, may still present limited repeatability. This finding highlights that surgical accuracy is a clinically relevant factor when evaluating the biological and biomechanical implications of reduced inter-implant distances [66,67].

More broadly, finite element analysis has played an important role in improving the understanding of how bone tissues respond to mechanical loading. An increasing number of studies have used different stress-analysis approaches to investigate the mechanical factors that may contribute to clinical complications or failure [66,67,68]. Although FEA cannot fully reproduce the complexity of in vivo conditions, it offers high repeatability and control of experimental variables, making it a valuable complementary tool for the study of implant biomechanics [69,70,71].

A three-dimensional FEA study by Simsek et al. [72] demonstrated that inter-implant distance has a significant influence on stress distribution in the bone surrounding endosseous implants in the posterior mandible. Specifically, the finite element method results showed that an inter-implant distance of 1.0 mm provided the most favorable stress distribution, with a more balanced compensation between tensile and compressive stresses in both cortical and trabecular bone. Shorter distances (0.5 mm) led to increased compressive stresses, mainly localized in the lingual region, whereas greater distances (2.0 mm) resulted in higher tensile stresses in the crestal area. However, such findings should be interpreted within the methodological limits of that computational setting and should not be directly extrapolated to clinical decision-making.

Similarly, the present study investigated the effect of inter-implant distance on bone density (D3 vs. D4) and implant position (crestal vs. subcrestal). The results revealed that the highest stress values were concentrated in the cortical bone surrounding the implants. For D3 bone, decreasing IID from 3 mm to 1 mm produced a progressive increase in cortical stress, from 7.2 MPa to 16.5 MPa, with higher values for crestally placed implants than for subcrestal ones. Despite this increase, all stresses remained within the mechanostat-based reference interval (2–20 MPa) adopted in the present study. Within this computational framework, this finding suggests a lower relative mechanical severity than configurations exceeding the upper threshold, but it does not exclude biologically unfavorable responses or clinically relevant overload.

For lower-quality D4 bone, cortical stresses were systematically higher and approached the upper threshold under crestal placement with an IID of 1 mm, indicating a less favorable mechanical condition within the adopted stress-interpretation framework.

These computational findings are not inconsistent with the clinical observations of Schwarz et al. [73]; however, no direct clinical inference can be drawn from the present model regarding the safety or acceptability of inter-implant distances below 3 mm.

Considering these observations, several limitations of the present study must be acknowledged. First, the mathematical model represents a simplification of clinical reality, for example, by assuming perfect osseointegration between implant and bone, a condition that is not always achieved in practice. Moreover, the loading and boundary conditions applied do not fully reproduce the complexity and variability of actual masticatory forces and individual anatomical differences. Finally, the absence of a prosthetic superstructure in the model, introduced to isolate the effect of inter-implant distance, may have significantly affected load transfer and stress distribution, thus limiting the direct translatability of the results to clinical scenarios in which prosthetic components play an important biomechanical role.

The results of the present study should be interpreted considering the simplifying assumptions of the numerical model, including perfect osseointegration, standardized material properties, and static loading conditions that do not fully reproduce the complexity of the in vivo biomechanical environment. Although the model is consistent with finite element approaches commonly adopted in implant biomechanics, its direct validation against experimental or clinical data remains limited. Therefore, the findings should be regarded as comparative preclinical computational evidence rather than direct clinical guidance, and further experimental and prospective clinical studies are needed to confirm their translational relevance.

## 5. Conclusions

The present finite element analysis showed that inter-implant distance influenced stress distribution in the surrounding peri-implant bone, with decreasing IID associated with higher cortical stress, particularly in crestally placed implants and in lower-density bone. Subcrestal placement was associated with lower cortical stress under the modeled conditions. Although all analyzed configurations remained within the mechanostat-based thresholds adopted in the present stress-interpretation framework, these findings should be regarded only as comparative computational results. No general conclusion can be drawn from the present model about the clinical acceptability or optimality of a 1 mm inter-implant distance. Further experimental and prospective clinical studies are required to determine the translational relevance of these observations.

## Figures and Tables

**Table 1:** Mechanical properties of bone.

Mechanical Properties	Cortical Bone	D3 Bone	D4 Bone
Young’s Modulus (E, MPa)	16000	1600	690
Poisson’s Ratio (v)	0.3	0.3	0.3

## Data Availability

The original contributions presented in this study are included in the article. Further inquiries can be directed to the corresponding author.
